# WHO target product profiles to shape global research and development

**DOI:** 10.2471/BLT.22.289521

**Published:** 2023-03-02

**Authors:** Sarah C Charnaud, Vasee Moorthy, John Reeder, Anna Laura Ross

**Affiliations:** a Research for Health Department, World Health Organization, Avenue Appia 20, 1211 Geneva 27, Switzerland.

## Abstract

Research and development leading to new and improved health products is essential for achieving healthier lives for populations worldwide. However, new products in development do not always match the global need for products for neglected diseases and populations. To promote research, provide an incentive for investment and align products with the needs of end-users, research needs to be better coordinated and prioritized. The World Health Organization (WHO) has developed target product profiles that define the characteristics required in new health products to address the greatest public health needs. A WHO target product profile document presents a need and provides guidance on what to include to consider access and equity as part of the research and development plan from the outset. WHO has also set up the Target Product Profile Directory, a free-to-use online database of characteristics used to describe desired health products, including medicines, vaccines, diagnostic tools and medical equipment. Here we describe the process of developing a WHO target product profile, and the benefits of this type of guidance. We urge product developers to share product profiles addressing unmet needs in public health, to help in progress towards global targets for better health and well-being.

## Résumé

Promouvoir la santé des populations à travers le monde va de pair avec la recherche-développement responsable de la conception et de l'optimisation de produits sanitaires. Pourtant, les nouveaux produits à l'étude ne répondent pas toujours aux exigences mondiales des populations et maladies négligées. En vue de promouvoir la recherche, de favoriser les investissements et d'aligner les produits sur les besoins des utilisateurs finaux, les travaux doivent être mieux coordonnés et leurs priorités, mieux définies. L'Organisation mondiale de la Santé (OMS) a donc élaboré des profils de produits cibles qui déterminent les caractéristiques requises pour les nouveaux produits sanitaires, afin qu'ils correspondent davantage aux besoins les plus criants en matière de santé publique. Un profil de produit cible établi par l'OMS est un document qui met en évidence un besoin et fournit des indications sur les aspects à prendre en compte pour garantir l'accès et l'équité dès le départ dans le plan de recherche-développement. L'OMS a également publié un Répertoire des profils de produits cibles, une base de données en ligne consultable gratuitement qui reprend les caractéristiques employées pour décrire les produits sanitaires souhaités (médicaments, vaccins, outils diagnostiques et équipements médicaux). Dans le présent document, nous détaillons le processus de développement d'un profil de produit cible par l'OMS, mais aussi les avantages que comportent de telles indications. Nous encourageons vivement les laboratoires à partager les profils de produits qui répondent à des besoins non satisfaits en matière de santé publique, afin de contribuer à avancer vers les objectifs mondiaux de santé et de bien-être.

## Resumen

La investigación y el desarrollo de productos sanitarios nuevos y mejorados son esenciales para que las poblaciones de todo el mundo vivan más sanas. Sin embargo, los productos nuevos en desarrollo no siempre se ajustan a las necesidades mundiales de productos para enfermedades y poblaciones desatendidas. Para promover la investigación, incentivar la inversión y adaptar los productos a las necesidades de los usuarios finales, es necesario coordinar mejor la investigación y establecer prioridades. La Organización Mundial de la Salud (OMS) ha elaborado perfiles de productos específicos que definen las características que deben reunir los productos sanitarios nuevos para satisfacer las principales necesidades de salud pública. Un documento de perfil de producto específico de la OMS presenta una necesidad y ofrece orientación sobre lo que debe incluirse para tener en cuenta el acceso y la equidad como parte del plan de investigación y desarrollo desde el principio. La OMS también ha creado el Directorio de Perfiles de Productos Específicos, una base de datos en línea de uso gratuito con las características utilizadas para describir los productos sanitarios deseados, incluidos medicamentos, vacunas, herramientas de diagnóstico y equipos médicos. En el presente documento, describimos el proceso de elaboración de un perfil de producto específico de la OMS y las ventajas de este tipo de orientación. Instamos a los desarrolladores de productos a compartir perfiles de productos que aborden necesidades no cubiertas en salud pública para contribuir al avance hacia los objetivos mundiales de mejora de la salud y el bienestar.

## ملخص

البحث والتطوير للمنتجات الصحية الجديدة والمحسنة أمران أساسيان لتحقيق حياة أكثر صحة للشعوب في كل أنحاء العالم. إلا أن المنتجات الجديدة قيد التطوير لا تتوافق دائمًا مع الحاجة العالمية للمنتجات المعنية بالأمراض والشعوب التي تلقى تجاهلاً. لتعزيز الأبحاث، وتوفير حافز للاستثمار واتساق المنتجات مع احتياجات المستخدمين النهائيين، تحتاج الأبحاث إلى شكل أفضل من التنسيق وتحديد الأولويات. لقد ابتكرت منظمة الصحة العالمية (WHO) أنماط المنتجات المستهدفة التي تحدد السمات المطلوبة في المنتجات الصحية الجديدة من أجل تلبية أكبر احتياجات الصحة العامة. إن مستند نمط المنتج المستهدف من منظمة الصحة العالمية يحدد احتياجًا ويقدم إرشادًا بشأن وضع التوفر والمساواة في الاعتبار ضمن خطة البحث والتطوير من بدايتها. كما أن منظمة الصحة العالمية قد أعدت دليلًا لنمط المنتج المستهدف، وهي قاعدة بيانات للاستخدام المجاني عبر الإنترنت تضم السمات المستخدمة في وصف المنتجات الصحية المرغوب بها، بما في ذلك الأدوية واللقاحات وأدوات التشخيص والمعدات الطبية. نحن نقدم هنا وصفًا لعملية ابتكار نمط منتج مستهدف من منظمة الصحة العالمية، ومزايا هذا النوع من الإرشاد. ونحث مطوري المنتجات على مشاركة أنماط المنتجات المعنية بالاحتياجات المطلوبة للصحة العامة، وذلك للمساعدة على التقدم نحو تحقيق الأهداف العالمية من أجل صحة ورفاهية أفضل.

## 摘要

研发新的和改进的医疗卫生产品对于全世界人民实现更健康的生活至关重要。然而，就被忽视的疾病和人群来说，正在开发的新产品并不总是符合其全球需求。为了促进研究，鼓励投资，并使产品符合最终用户的需求，需要更好地协调研究并确定研究的优先次序。世界卫生组织 (WHO) 制定了目标产品简介，确定了新医疗卫生产品所需的特征，以满足最大的公共卫生需求。世卫组织目标产品简介文件提出了一种需求，并就从一开始将可及性和公平性作为研发计划的一部分考虑所应包括的内容提供了指导。世卫组织还建立了目标产品简介目录，这是一个用于描述所需医疗卫生产品（包括药物、疫苗、诊断工具和医疗设备）的在线数据库，可供免费使用。在此，我们描述了制定世卫组织目标产品简介的过程以及此类指导的好处。我们敦促产品开发人员分享解决公共卫生方面未满足需求的产品简介，以帮助实现增进健康和福祉的全球目标。

## Резюме

Научные исследования и разработка новых и усовершенствованных изделий для медицинского назначения необходимы для поддержания здорового образа жизни населения во всем мире. Однако разрабатываемые новые продукты не всегда соответствуют глобальной потребности в продуктах для лечения забытых болезней и игнорируемых популяций. Для содействия проведению научных исследований, создания стимулов для инвестиций и приведения продукции в соответствие с потребностями конечных пользователей необходимо лучше координировать исследования и расставлять приоритеты. Всемирная организация здравоохранения (ВОЗ) разработала профили целевых продуктов, которые определяют характеристики, необходимые новым изделиям для медицинского назначения для удовлетворения важнейших потребностей общественного здравоохранения. Документ ВОЗ с описанием профиля целевого продукта отражает потребность и содержит рекомендации относительно того, что необходимо включить, чтобы с самого начала учитывать вопросы доступности и равных возможностей при составлении плана научных исследований и разработок. ВОЗ также разработала Справочник целевых профилей продукции (Target Product Profile Directory) – бесплатную для использования онлайн-базу данных характеристик, используемых для описания желаемых изделий для медицинского назначения, включая лекарственные препараты, вакцины, диагностические инструменты и медицинское оборудование. В нем описан процесс разработки профиля целевого продукта ВОЗ, а также преимущества такого рода руководства. Мы призываем разработчиков делиться профилями продукции, указывающими на востребованность в области общественного здравоохранения, в целях достижения глобальных целей по укреплению здоровья и поддержания здорового образа жизни.

## Introduction

In the past two decades, invaluable global health gains have been achieved through the development and scale-up of new and improved health products,^1^ resulting in lives saved and healthier populations. Despite significant progress in the prevention and treatment of communicable diseases, there are still unmet needs.^2^ The burden of noncommunicable diseases too is increasing. In 2019, the World Health Organization (WHO) set the ambitious Triple Billion targets for 2023, calling for one billion more people to enjoy better health and well-being, one billion more people to be better protected from health emergencies, and one billion more people to benefit from universal health coverage.^3^

Targeted and prioritized research and development of new health products can be an essential step towards these goals. The process of research and development of new products is currently fragmented. Differing priorities among researchers, funders and the people for whom the intervention is intended can lead to the development of products that are ill-suited, or even unavailable, to the people who need them. For example, a product might be designed and tested for use in laboratory settings rather than remote settings, for use by highly trained professionals rather than community health workers, or not be easily transportable to where it is needed. The difficulties are exacerbated by limited funding for new products targeting the needs of people in lower-income countries. A WHO report in 1999 highlighted that only 10% of the world’s spending on research and development focused on the health concerns affecting 90% of the population.^4^ This so-called 10/90 gap has not significantly improved since the report, partly due to the lack of incentives for research and development of health products affecting populations in under-resourced settings.

Different solutions have been developed to improve the coordination of research and development to address the world’s most pressing public health needs. The development of proposals and plans provide clear approaches to coordinate and prioritize research and development to meet global health needs. Research and development plans generally require clear indications of the characteristics that are needed in a new health product.^5^

A tool already exists to shape and guide global health product development. Target product profiles are documents that indicate the characteristics that are most desirable in a specific health product. The aim is to coordinate and incentivize research and development to accelerate development of products that are fit-for-purpose. The target product profile approach was first used to enable discussion internally within a company to discuss technical products, and later became more commonly used to facilitate discussions between the pharmaceutical industry and regulators.^6^ Target product profiles are increasingly used in public health. The use of target product profiles at the development stage appears to accelerate regulatory approval. A study of submissions to the United States Food and Drug Administration found that those that referenced a target product profile were approved around 10% faster than those that did not.^6^ Using target product profiles to align with research and development needs should lead to better products and faster development of products.

## WHO target product profiles

### Principles

Target product profiles developed by WHO are intended to support the development of products for neglected diseases and populations, including preventive methods (for example, vaccines or vector control), therapeutic tools, diagnostic tools and medical devices. A WHO target product profile document should inform product developers, regulatory agencies, procurement agencies and funders about both research and development needs and public health priorities. Development of a WHO target product profile recognizes that access, equity and affordability are integral parts of the process and need to be considered from the earliest stages of development. 

A WHO target product profile: (i) indicates a major public health need for the product, and provides an incentive for research, development and manufacture by indicating that such a product would be welcomed; (ii) considers end-user perspectives from the outset, and facilitates alignment among stakeholders on what will constitute a product that is fit-for-purpose; (iii) considers access and equity from the outset, supporting ethical research and development of the product; and (iv) helps to convene the individuals and organizations involved in research to reach a consensus on the required product characteristics. 

A product that meets these requirements can be reviewed more easily to accelerate the process of developing guidelines. 

### Content

The type of guidance provided by WHO may differ depending on the stage of product development. The term *target product profile* refers to a document containing: (i) the optimal characteristics required for implementation of a health product; and (ii) the minimal characteristics below which it is unlikely a product would be useful. A study found that WHO target product profiles were most useful to manufacturers for understanding how, where and by whom a product should be used.^7^

Where WHO has identified a priority need for a product class but development is at an early stage, the term *preferred product characteristics* may be used. These documents focus on providing strategic guidance and higher-level considerations and only specify WHO’s preferences. For some diseases the important issues are how therapeutics can be best combined into treatment regimens, for example, in tuberculosis. In this case, target product profiles have been framed as the *target regimen profile*. We use the term *target product profile* throughout this document, and in general it is intended to be used interchangeably with preferred product characteristics and target regimen profile.

### Objectives

The WHO target product profile document is intended to facilitate fast and efficient development of products addressing the greatest and most urgent public health needs. Target product profiles drive innovation by providing information for funders and developers as to the public health perspective on the expected use and characteristics of products being developed (such as the cost, level of training required, duration of use, and storage and working conditions). WHO target product profiles should be considered as guidance at all stages of the process – linking considerations around product development, access and affordability of products, regulatory issues and policy goals – so that product development can proceed with public health goals in mind. The WHO Science Division supports the development of the highest quality target product profiles.

## WHO Directory

To increase awareness of target product profiles^7^ and to improve the efficiency of efforts to develop new products, WHO has developed a searchable directory of target product profiles.^8^ The Target Product Profile Directory is a free-to-use online database of characteristics used to describe desired health products, including medicines, vaccines, diagnostic tools and medical equipment. The directory provides links to access the full product profile document.

The directory was developed in response to the Ebola virus disease outbreak of 2013–2016, which highlighted the urgent need for coordination in research efforts. The directory provides a repository for existing health product profiles, and introduces harmonization and standardization in the description of health product profiles. The directory also enables a high-level analysis of research and development activity related to these product profiles. Finally, the directory emphasizes access, equity and affordability as integral parts of the innovation process that need to be considered at all stages, not after a product has been developed.

Any organization that develops a target product profile that meets the inclusion criteria can submit a summary of their product for inclusion in the directory. Inclusion in the directory is not an endorsement of a specific set of product characteristics, but rather an opportunity to collate documents in one place and align research and development efforts to produce the most-needed public health products. While WHO teams have been developing, updating or adapting target product profiles and submitting them to the directory, there has been limited submissions of target product profiles from other organizations in the last two years. This gap may be due to limited awareness about the directory among individuals and organizations, some submissions providing insufficient information and thus being unsuitable for publication in the directory, and by the directory being offline for an extended period. We urge all target product profile developers to share product profiles addressing unmet needs in global public health.

## Health impacts

**Fig. 1:**
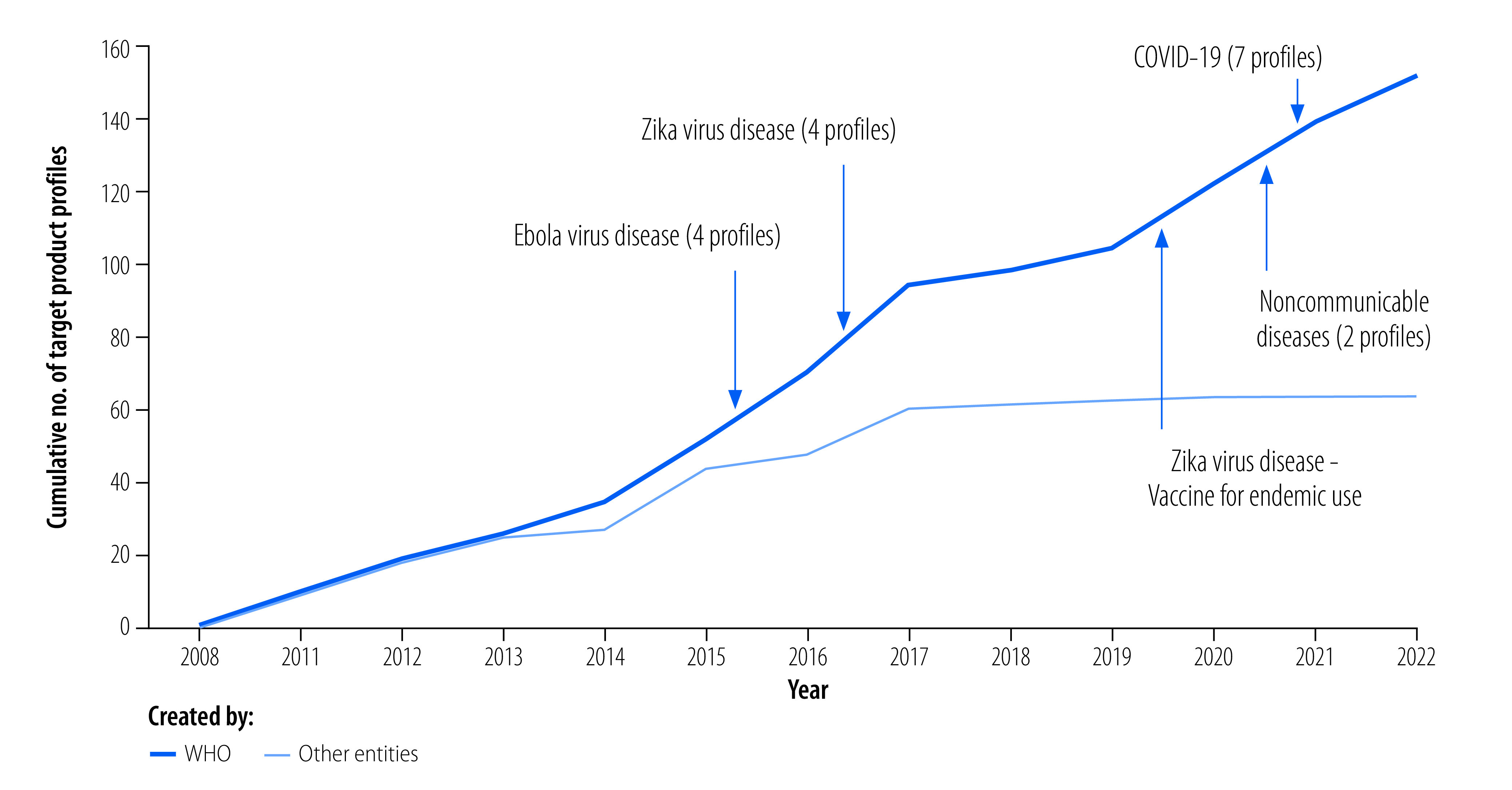
Target product profiles published in the WHO directory, 2008–2022

In recent public health emergencies, target product profiles have been an important tool to align with research and development needs and to rapidly develop the most suitable products.^9^ Target product profiles were developed to guide the development of diagnostic tools and vaccines for the coronavirus disease 2019 (COVID-19), Ebola virus disease and Zika virus outbreaks (Fig. 1). The product profile on Ebola virus vaccines focused on the time from first vaccine dose to the development of immunity so that ring vaccination could be used to rapidly control outbreaks by targeting those at increased risk. Target product profiles are also regularly used for research and development of health products to combat many endemic diseases such as malaria, neglected tropical diseases and tuberculosis.^10^ A target product profile for pneumococcal vaccines provided important guidance on the serotypes to be included in vaccines to meet the needs of low- and middle-income countries. Analysis of whether and how target product profiles influence the development of products that are fit-for-purpose could provide insights into the optimal development of a target product profile.

WHO: World Health Organization.

Note: The chart shows the cumulative number of target product profiles published in the WHO Target Product Profile Directory by date of development of the profile. The labels indicate the timeline of important target product profiles developed for public health emergencies and non-emergencies.

Data source: Target Product Profile Directory, WHO.^8^

Historically, most target product profiles developed by WHO have focused on infectious diseases. There is currently an increase in the development of product profiles for noncommunicable diseases, focusing on the characteristics needed in products for low- and middle-income countries. An example is the product profile for therapy for respiratory distress syndrome in neonates.^11^ We see great opportunities for research and development of products for chronic conditions, particularly for populations with unmet needs.

In summary, target product profiles and similar documents can outline public health needs from the perspective of users, and consider issues of access and equity at all stages of product development. The publication of WHO target product profiles focuses attention on needed products and accelerates the development process. Used well, target product profiles can guide research and development for products to achieve better health for people in areas where there are significant unmet needs in global health.
